# Factors of the Nursing Practice Environment Shaping Nurses’ Perceived Benefits of Adverse Event Reporting: A Cross-Sectional Study Among Primary Healthcare Nurses

**DOI:** 10.3390/healthcare14121727

**Published:** 2026-06-16

**Authors:** Kuralai Utzhanova, Gulshara Aimbetova, Dinara Makhanbetkulova, Aurelija Blazeviciene, Nargiza Nassyrova, Akmaral Khalelova, Aizat Aimakhanova, Zhenis Mukhamedkerim

**Affiliations:** 1 Department of Nursing, Kazakh National Medical University, Almaty 050012, Kazakhstan; utzhanova75@mail.ru (K.U.); mukhamedkerim.z@gmail.com (Z.M.); 2 Department of Public Health, Asfendiyarov Kazakh National Medical University, Almaty 050012, Kazakhstan; aimbetova.g@kaznmu.kz; 3 Department of Nursing, Faculty of Nursing, Lithuanian University of Health Sciences, 50161 Kaunas, Lithuania; aurelija.blazeviciene@lsmu.lt; 4 Department of Scientific Research, Kazakh-Russian Medical University, Almaty 050012, Kazakhstan; nassyrova.n94@gmail.com; 5 Department of Nursing, Higher Medical College, Almaty 050012, Kazakhstan; akow_0390@mail.ru; 6 Department of Biostatistics and Basics of Scientific Research, Asfendiyarov Kazakh National Medical University, Almaty 050012, Kazakhstan; aimahanova.a@kaznmu.kz

**Keywords:** patient safety, adverse event reporting, nursing practice environment, incident reporting, nurses, primary healthcare

## Abstract

**Background:** Adverse event reporting is a critical component of patient safety systems; however, nurses’ engagement in reporting is influenced not only by reporting procedures but also by broader organizational characteristics of the nursing practice environment. Although previous studies have examined reporting behaviors in various healthcare settings, limited evidence is available regarding how organizational factors influence nurses’ perceptions of adverse event reporting in post-Soviet primary healthcare systems. **Objective:** To examine the relationship between the nursing practice environment and nurses’ perceived benefits of adverse event reporting in primary healthcare settings in Kazakhstan and to explore the underlying factor structure of the nursing practice environment within this context. **Methods:** A cross-sectional survey was conducted among 468 primary healthcare nurses from six major cities in Kazakhstan. Participants were recruited through professional and educational networks using a targeted convenience sampling strategy. The nursing practice environment was assessed using the Revised Professional Practice Environment (RPPE) scale, while attitudes toward adverse event reporting were measured using the Reporting of Clinical Adverse Events Scale (RoCAES), focusing on the perceived benefits of reporting dimension. Exploratory factor analysis was performed to identify the underlying structure of the RPPE scale. Associations between EFA-derived factors and perceived benefits of adverse event reporting were examined using Spearman correlation analysis and multivariable logistic regression with adjustment for age, gender, city, and professional position. **Results:** Exploratory factor analysis identified three dimensions of the nursing practice environment: Professional Motivation and Teamwork, Interprofessional Conflict and Workplace Relationships, and Staffing Adequacy. Spearman correlation analysis demonstrated significant associations between all three factors and perceived benefits of adverse event reporting. Factor 1 (Professional Motivation and Teamwork) showed the strongest negative correlation with the outcome (r = −0.562, *p* < 0.001), followed by Factor 3 (Staffing Adequacy) (r = −0.434, *p* < 0.001), whereas Factor 2 (Interprofessional Conflict and Workplace Relationships) demonstrated a positive correlation (r = 0.227, *p* < 0.001). In the multivariable logistic regression model adjusted for age, gender, city, and professional position, Factor 1 was negatively associated with favorable perceptions of adverse event reporting (OR = 0.389, *p* < 0.001), whereas Factor 2 demonstrated a positive association (OR = 1.763, *p* = 0.002). Factor 3 and demographic variables were not statistically significant. **Conclusions:** The findings suggest that nurses’ perceptions of the benefits of adverse event reporting are influenced by multiple dimensions of the nursing practice environment. Exploratory factor analysis identified three organizational dimensions—Professional Motivation and Teamwork, Interprofessional Conflict and Workplace Relationships, and Staffing Adequacy—that were associated with reporting perceptions. After adjustment for demographic characteristics, Professional Motivation and Teamwork and Interprofessional Conflict and Workplace Relationships remained independently associated with perceived benefits of adverse event reporting, whereas demographic factors did not demonstrate significant associations. These findings highlight the importance of organizational conditions, communication processes, and professional engagement in shaping nurses’ attitudes toward adverse event reporting. Efforts to strengthen patient safety reporting systems should therefore extend beyond reporting procedures alone and include broader organizational strategies aimed at improving communication, teamwork, and supportive work environments within primary healthcare settings.

## 1. Introduction

Patient safety continues to be a major priority for healthcare systems worldwide, as adverse events remain a significant source of preventable harm, increased healthcare costs, prolonged hospital stays, and reduced public trust in health services [1,2]. The capacity of healthcare organizations to identify, report, and learn from such events is widely regarded as a fundamental component of patient safety culture and quality improvement initiatives [3,4]. Nurses play a central role in this process, as they provide continuous patient care and are often the first to recognize clinical deterioration, near misses, and unsafe practices [5,6].

Despite the recognized importance of adverse event reporting, underreporting remains a persistent challenge across healthcare systems [7,8]. A range of organizational and psychological barriers may discourage reporting, including fear of blame, uncertainty regarding reporting criteria, lack of feedback, and unclear reporting procedures [9,10,11]. As a result, reporting behavior is shaped not only by individual attitudes but also by the broader organizational and professional context in which nurses operate [12].

The nursing professional practice environment encompasses organizational characteristics that support or constrain nursing practice, including leadership, autonomy, communication, teamwork, interprofessional relationships, staffing adequacy, and institutional support [13,14]. A supportive practice environment has been consistently linked to improved job satisfaction, reduced burnout, better staff retention, and enhanced patient outcomes [15,16,17]. Moreover, it may shape patient safety behaviors by influencing communication processes, perceived responsibility, and the perceived value of reporting systems [18].

The study was conceptually informed by organizational and patient safety frameworks emphasizing the interaction between professional work environments and safety-related attitudes and behaviors.

This conceptual approach is broadly consistent with organizational behavior and Job Demands–Resources (JD-R) perspectives, which propose that workplace conditions influence professional attitudes, motivation, and safety-related behaviors. Within healthcare settings, organizational resources such as communication, teamwork, leadership support, and adequate staffing may shape how nurses perceive the usefulness, value, and organizational importance of adverse event reporting systems. Consequently, perceptions of reporting are viewed not solely as individual attitudes but also as reflections of broader organizational environments in which patient safety practices are embedded.

However, the relationship between the practice environment and adverse event reporting is complex and may vary across organizational contexts. In some contexts, supportive environments may encourage engagement with formal reporting systems. In other contexts, safety concerns may also be addressed through informal communication and local problem-solving processes, which could influence engagement with formal reporting procedures [19,20].

Existing studies have primarily examined adverse event reporting in relation to patient safety culture, reporting barriers, and organizational climate. In parallel, research on the nursing practice environment has predominantly focused on outcomes such as job satisfaction, burnout, and quality of care [21,22,23,24,25]. However, limited attention has been given to the intersection of these domains, particularly regarding how organizational dimensions of the nursing practice environment influence nurses’ perceptions of the benefits of adverse event reporting.

This gap is particularly relevant in Central Asian and post-Soviet healthcare systems, where organizational structures, hierarchical dynamics, and the evolution of nursing roles differ from those described in Western contexts [26,27]. Consequently, empirical evidence from these settings remains scarce, and current literature provides limited insight into how organizational factors shape reporting perceptions in these environments.

Addressing this gap is important for both theoretical and practical reasons. From a theoretical perspective, examining these relationships contributes to a more integrated understanding of patient safety behaviors within organizational contexts. From a practical perspective, identifying organizational factors associated with more favorable perceptions of adverse event reporting may inform the development of targeted strategies to strengthen patient safety culture and improve reporting systems. In particular, nurses are more likely to engage in reporting when they perceive reporting systems as meaningful, useful, and supportive of clinical improvement [28].

Therefore, the present study aimed to examine how characteristics of the nursing professional practice environment are associated with nurses’ perceptions of the benefits of adverse event reporting in primary healthcare settings in Kazakhstan. In addition, the study explored the underlying factor structure of the Revised Professional Practice Environment (RPPE) scale to examine how dimensions of the nursing practice environment are organized within the local healthcare context.

Based on previous organizational behavior and patient safety literature, we hypothesized that organizational dimensions of the nursing practice environment would be associated with nurses’ perceptions of the benefits of adverse event reporting, reflecting the influence of workplace conditions on safety-related attitudes and behaviors.

The study was guided by the following research question: Which organizational dimensions of the nursing practice environment are associated with nurses’ perceived benefits of adverse event reporting in primary healthcare settings?

## 2. Materials and Methods

### 2.1. Study Design

A descriptive cross-sectional approach was used in this study. The reporting of the study followed the recommendations of the Strengthening the Reporting of Observational Studies in Epidemiology (STROBE) guidelines.

### 2.2. Setting and Sample

The study population consisted of registered nurses employed in public primary healthcare (PHC) settings across six major cities in Kazakhstan, including Almaty, Astana, Shymkent, Karaganda, Semey, and Aktobe.

Data collection was carried out using an online questionnaire distributed through six medical universities located in these cities. The survey link was disseminated via professional and academic communication channels to nurses enrolled in accelerated evening training programs who were concurrently employed in PHC settings.

Participants were informed prior to participation that the survey was intended only for nurses currently working in PHC. In addition, the questionnaire included a screening item on workplace setting, and only responses confirming employment in PHC were retained for analysis.

A targeted convenience sampling strategy was applied through professional, academic, and educational networks. Participants were recruited through professional and educational communication networks associated with six medical universities, including nurses enrolled in accelerated evening educational programs while concurrently employed in primary healthcare settings.

Healthcare organizations were not used as direct sampling units, as recruitment was conducted through professional and educational communication channels rather than through institutional selection procedures. Consequently, the study sample should not be interpreted as nationally representative of all primary healthcare nurses in Kazakhstan.

The sampling strategy may have resulted in a higher representation of nurses actively involved in continuing education, academic activities, and professional development. Therefore, the findings are primarily applicable to nurses participating in professional and educational networks within urban public primary healthcare settings and should not be interpreted as representative of all PHC nurses in Kazakhstan.

The recruitment strategy aimed to include all accessible eligible participants available during the data collection period through targeted professional and educational networks. The final sample size (*n* = 468) was considered sufficient for the planned statistical analyses, including exploratory factor analysis and multivariable logistic regression modeling, and exceeded commonly recommended minimum sample size recommendations for factor analytic procedures.

Participant eligibility was defined based on predefined inclusion and exclusion criteria.

Inclusion criteria were:

(1) Registered nurses working in primary healthcare settings;
(2) Active involvement in clinical practice;
(3) Willingness to participate in the study.

Exclusion criteria were:

(1) Incomplete questionnaire responses;
(2) Respondents not employed in PHC settings;
(3) Nurses on leave during the data collection period.

The study protocol was reviewed and approved by the Local Ethics Committee of S.D. Asfendiyarov Kazakh National Medical University (Approval No. 04-101224, 29 April 2025). Participation was voluntary and anonymous, and informed consent was obtained from all participants before completing the survey.

### 2.3. Instruments

Two previously validated instruments were utilized in this study.

Before data collection, both instruments were translated into Kazakh and Russian following a forward–backward translation procedure. The initial translation was carried out by faculty members of the Department of Nursing at S.D. Asfendiyarov Kazakh National Medical University with both academic and clinical expertise. This was followed by an independent back-translation into English to verify conceptual and linguistic consistency with the original versions. Any discrepancies identified during this process were reviewed and resolved through consensus among the research team. These steps were undertaken to ensure conceptual, semantic, and cultural equivalence of the instruments.

In the current study, selected psychometric properties of the instruments were evaluated, including internal consistency and underlying factor structure. Full psychometric validation of the instruments within the Kazakhstani context was beyond the scope of the present research.

#### 2.3.1. Nursing Practice Environment

The nursing professional practice environment was evaluated using the Revised Professional Practice Environment (RPPE) scale [29]. This instrument was chosen due to its ability to capture a broad range of organizational and relational aspects of the practice environment. The RPPE includes 42 items grouped into eight subscales: leadership and autonomy in clinical practice, control over practice, communication about patients, teamwork, handling disagreement and conflict, internal work motivation, staff relationships with physicians, and cultural sensitivity.

All items were rated on a 4-point Likert scale ranging from 1 (strongly disagree) to 4 (strongly agree), with higher scores reflecting more favorable perceptions of the professional practice environment. In the present study, the total RPPE scale demonstrated high internal consistency (Cronbach’s α = 0.87).

The RPPE assesses nurses’ perceptions of the professional practice environment rather than objective organizational characteristics. Although previous studies have demonstrated acceptable structural validity and reliability of the instrument, criterion validity has not yet been extensively examined. Therefore, the present study should be interpreted as examining associations among nurses’ self-reported perceptions of their work environment and their perceptions of adverse event reporting rather than objective organizational conditions.

#### 2.3.2. Attitudes Toward Adverse Event Reporting

Nurses’ attitudes toward adverse event reporting were evaluated using the Reporting of Clinical Adverse Events Scale (RoCAES) [30]. This instrument was selected as it is specifically designed to assess healthcare professionals’ perceptions and attitudes related to adverse event reporting and has been widely applied in patient safety research.

The RoCAES consists of 25 items organized into five domains: perceived blame associated with reporting, criteria for identifying reportable events, perceptions of colleagues’ expectations, perceived benefits of reporting, and clarity of reporting procedures.

All items were rated using a 4-point Likert scale. Higher scores on the “perceived benefits of reporting” subscale reflect more positive perceptions of the usefulness and value of adverse event reporting.

Prior to analysis, negatively worded items were reverse-coded to ensure consistency in score interpretation, with higher values indicating more positive attitudes. In this study, the total RoCAES scale demonstrated satisfactory internal consistency (Cronbach’s α = 0.84).

#### 2.3.3. Demographic Characteristics

Information on participants’ demographic and professional background was collected, including age, gender, clinical experience, qualification category, and level of nursing education.

### 2.4. Data Collection

Data collection was conducted between 15 September and 20 November 2025 using an anonymous online questionnaire administered through Google Forms (Google LLC, Mountain View, CA, USA) [31]. The survey link was disseminated via professional and academic communication channels to reach nurses who met the inclusion criteria.

Participants were informed prior to participation that the survey was intended exclusively for nurses working in primary healthcare settings. Before accessing the questionnaire, respondents were provided with an electronic informed consent statement describing the study objectives, the voluntary nature of participation, data confidentiality, and the right to withdraw at any stage. Proceeding to the questionnaire was considered as provision of informed consent.

No personally identifiable information, such as names, contact details, or IP addresses, was collected. All data were stored in a secure, password-protected database accessible only to members of the research team.

Participation was voluntary, and no incentives were offered. While these measures were implemented to reduce potential response bias, the possibility of social desirability bias inherent in self-reported data cannot be entirely excluded.

### 2.5. Data Preparation

Prior to analysis, negatively worded items were reverse-coded so that higher scores consistently represented more positive perceptions.

The dataset was examined for missing values and inconsistencies. Questionnaires with substantial missing data were excluded according to predefined data-cleaning criteria. No imputation procedures were applied, and all analyses were performed using complete-case data.

Mean scores were calculated for each scale and subscale. For descriptive purposes and for binary logistic regression analysis, responses were categorized using a cut-off value of 2.5, corresponding to the midpoint of the 4-point Likert scale. Values below 2.5 were interpreted as less favorable perceptions, whereas values of 2.5 or higher indicated more favorable perceptions [32].

Likert-scale variables were treated as continuous for descriptive and correlational analyses to preserve variability and better capture the strength and direction of associations.

### 2.6. Statistical Analysis

All statistical analyses were performed using IBM SPSS Statistics for Windows, version 29.0 (IBM Corp., Armonk, NY, USA).

Descriptive statistics were applied to summarize participants’ characteristics and study variables. Continuous variables are presented as means with standard deviations, whereas categorical variables are described using frequencies and percentages.

The internal consistency of the measurement instruments was evaluated using Cronbach’s alpha coefficients.

To explore the underlying structure of the instruments within the study sample, exploratory factor analysis (EFA) was performed using principal axis factoring with Varimax rotation. This procedure was intended to assess the structural consistency of the instruments rather than to conduct a full psychometric validation. The suitability of the data for factor analysis was examined using the Kaiser–Meyer–Olkin (KMO) measure and Bartlett’s test of sphericity. Factor loadings of 0.40 or higher were considered significant for interpretation.

Associations between variables were assessed using Spearman’s rank correlation coefficient due to the ordinal nature of the data.

To identify independent predictors of perceived benefits of adverse event reporting, multivariable binary logistic regression analysis was conducted. Prior to model estimation, key assumptions were evaluated, including multicollinearity, which was assessed using variance inflation factors (VIF). All VIF values were within acceptable limits.

For logistic regression analysis, scores ≥ 2.5 on the perceived benefits subscale were coded as “1” (more favorable perceptions of adverse event reporting), whereas scores < 2.5 were coded as “0” (less favorable perceptions).

Exploratory factor analysis was conducted to examine the underlying organizational structure of the RPPE scale within the local study context. Because the analysis identified a contextually interpretable factor structure, EFA-derived factors were subsequently used in correlation and regression analyses. The original RPPE subscales were retained for descriptive interpretation and comparison with the existing literature, as they represent previously validated and conceptually established dimensions of the professional practice environment. The EFA was used primarily to explore latent organizational patterns and assess contextual structural consistency of the RPPE scale within the local healthcare setting rather than to develop a new measurement model.

Factors with eigenvalues > 1.0 were initially retained for consideration. Items with factor loadings ≥ 0.40 were considered meaningful. Factor retention was further informed by scree plot inspection, conceptual interpretability, and evaluation of cross-loadings. Factors demonstrating substantial cross-loadings or limited conceptual coherence were not retained for further interpretation.

The midpoint cut-off was selected for interpretive purposes and to facilitate binary logistic regression analysis, consistent with approaches used in previous Likert-scale studies. This threshold corresponded to the conceptual midpoint of the 4-point Likert scale and was applied for interpretive purposes rather than diagnostic classification.

A *p*-value of less than 0.05 was considered statistically significant.

## 3. Results

### 3.1. Participant Characteristics

Table 1 summarizes the demographic profile of the study participants. The final sample included 468 nurses employed in public primary healthcare settings across six major cities in Kazakhstan (Almaty, Astana, Shymkent, Aktobe, Semey, and Karaganda).

Participants’ ages ranged from 20 to 58 years, with a mean age of 38.5 years (SD = 10.68). The duration of professional experience varied from 1 year to over 25 years, with an average of 12.5 years (SD = 8.2).

The sample was overwhelmingly female (97.2%, *n* = 455), while male participants accounted for 2.8%. This distribution is consistent with the gender composition typically observed in the nursing workforce.

In terms of clinical roles, most respondents were staff nurses (85.0%), followed by senior nurses (12.8%) and head nurses (2.1%).

Overall, the study population predominantly comprised mid-career female nurses working in clinical roles within urban primary healthcare settings.

### 3.2. Reliability of the Study Instruments

The internal consistency of the instruments is presented in Table 2.

The Revised Professional Practice Environment (RPPE) scale demonstrated high reliability, with a Cronbach’s alpha of 0.87. The Reporting of Clinical Adverse Events Scale (RoCAES) also showed good internal consistency, with a Cronbach’s alpha of 0.84.

Both values exceed the commonly accepted threshold of 0.70, indicating satisfactory reliability of the instruments within the study sample.

Both instruments demonstrated acceptable overall internal consistency within the study sample.

### 3.3. Factor Structure of the RPPE Scale

An exploratory factor analysis (EFA) was performed to identify the latent structure of the Revised Professional Practice Environment (RPPE) scale within the study sample.

The adequacy of the dataset for factor analysis was confirmed by a high Kaiser–Meyer–Olkin (KMO) value of 0.949, indicating excellent sampling adequacy. In addition, Bartlett’s test of sphericity was statistically significant (χ^2^ = 12,665.842; df = 861; *p* < 0.001), supporting the presence of sufficient correlations among variables to justify factor extraction.

Factor extraction was carried out using principal axis factoring, followed by orthogonal Varimax rotation with Kaiser normalization. The initial solution yielded six factors with eigenvalues exceeding 1.0, collectively explaining 56.98% of the total variance.

Despite meeting the statistical criteria, the six-factor solution demonstrated limited conceptual clarity due to cross-loadings and overlapping item distributions. Although six factors initially met the Kaiser criterion (eigenvalue > 1.0), examination of the scree plot, factor interpretability, and cross-loading patterns supported retention of a more parsimonious three-factor solution for subsequent analyses.

Before rotation, the first factor accounted for 36.15% of the variance, while the second factor explained 10.56%. The remaining factors contributed smaller proportions of explained variance (3.62%, 2.75%, 2.45%, and 1.45%, respectively). Following rotation, variance was redistributed across factors, resulting in a more balanced structure (21.40%, 10.90%, 7.42%, 7.12%, 6.47%, and 3.69%).

Items with factor loadings of 0.40 or higher were considered to contribute meaningfully to factor interpretation. Most items demonstrated communalities above 0.40, indicating that the extracted factors adequately represented the underlying variance of the RPPE scale.

The resulting factor structure differs from previously reported RPPE configurations, reflecting context-specific organizational patterns within the study setting. Because the exploratory factor analysis identified a conceptually coherent three-factor structure, the EFA-derived factors were used in subsequent correlation and regression analyses.

The three-factor solution was retained based on interpretability, conceptual coherence, factor loading patterns, and evaluation of cross-loadings.

The rotated factor loading matrix is presented in Table 3.

The labeling of factors was based on the conceptual similarity of items with the highest factor loadings within each factor. Based on the pattern of item loadings, the extracted factors were interpreted as representing professional motivation and teamwork, Interprofessional Conflict and Workplace Relationships, and staffing adequacy.

Inspection of the rotated factor matrix indicated that the majority of meaningful item loadings were concentrated within three interpretable factors. The remaining factors contained only a small number of items with weak or cross-loadings and were therefore not retained for further interpretation. Thus, the three-factor structure was retained for exploratory interpretation.

The identified three-factor structure may reflect key organizational dimensions of the professional practice environment within the local study context.

### 3.4. Associations Between Nursing Practice Environment Factors and Perceived Benefits of Adverse Event Reporting

Spearman correlation analysis was conducted to examine the associations between the EFA-derived factors of the nursing practice environment and perceived benefits of adverse event reporting. As shown in Table 4, all three factors demonstrated statistically significant correlations with the outcome variable.

Factor 1 (Professional Motivation and Teamwork) showed the strongest negative correlation with perceived benefits of adverse event reporting (r = −0.562, *p* < 0.001), indicating that higher scores on this factor were associated with less favorable perceptions of adverse event reporting benefits. Factor 3 (Staffing Adequacy) also demonstrated a moderate negative correlation with the outcome (r = −0.434, *p* < 0.001).

In contrast, Factor 2 (Interprofessional Conflict and Workplace Relationships) demonstrated a weak positive correlation with perceived benefits of adverse event reporting (r = 0.227, *p* < 0.001), suggesting that higher scores on this factor were associated with more favorable perceptions of reporting benefits.

Overall, the findings indicate that the relationships between nursing practice environment factors and perceived benefits of adverse event reporting vary across dimensions of the work environment, underscoring the multidimensional nature of these associations and supporting their inclusion in subsequent multivariable analyses.

### 3.5. Predictors of Perceived Benefits of Adverse Event Reporting: Logistic Regression Analysis

For logistic regression analysis, the perceived benefits subscale of the RoCAES instrument was dichotomized using the conceptual midpoint value of 2.5. Scores ≥ 2.5 were coded as “1” (more favorable perceptions of adverse event reporting), whereas scores < 2.5 were coded as “0” (less favorable perceptions). Based on the dichotomized outcome variable, 378 participants (80.9%) demonstrated less favorable perceptions (<2.5), whereas 89 participants (19.1%) demonstrated more favorable perceptions (≥2.5). One case with missing outcome data was excluded from logistic regression analysis.

The Hosmer–Lemeshow test indicated adequate model fit (χ^2^ = 7.64, df = 8, *p* = 0.470). To address potential confounding, the logistic regression model was adjusted for participants’ age, city, gender, and professional position. Factor 1 was negatively associated with favorable perceptions of adverse event reporting (OR = 0.389, *p* < 0.001), whereas Factor 2 was positively associated with the outcome (OR = 1.763, *p* = 0.002). Factor 3 was not statistically significant (OR = 0.751, *p* = 0.157). None of the demographic variables were significantly associated with the outcome. Importantly, adjustment for demographic characteristics did not materially alter the observed associations between the EFA-derived factors and perceived benefits of adverse event reporting. The model demonstrated moderate explanatory power (Nagelkerke R^2^ = 0.329).

The results of the logistic regression analysis are presented in Table 5.

The model was additionally adjusted for city (overall *p* = 0.192) and position (overall *p* = 0.865), neither of which was significantly associated with the outcome.

## 4. Discussion

This study examined the relationship between characteristics of the nursing professional practice environment and nurses’ perceptions of the benefits of adverse event reporting in primary healthcare settings across six major cities in Kazakhstan. In addition, the study explored the underlying structure of the RPPE scale within the local healthcare context using exploratory factor analysis.

The findings demonstrated that the nursing practice environment represents a multidimensional organizational construct associated with nurses’ perceptions of adverse event reporting. Exploratory factor analysis identified three interpretable dimensions of the professional practice environment: professional motivation and teamwork, Interprofessional Conflict and Workplace Relationships, and staffing adequacy. Although the original RPPE instrument conceptualizes the nursing practice environment through multiple subscales, the present findings suggest that, within the studied context, several organizational and interpersonal characteristics cluster into broader organizational domains. Similar multidimensional patterns have been reported in previous studies examining nursing work environments and their influence on quality of care, staff outcomes, and patient safety [33,34].

The identified factor structure may reflect specific organizational characteristics of healthcare systems undergoing professional and structural transformation. In Kazakhstan, nursing roles continue to evolve within healthcare organizations that have historically been characterized by hierarchical management structures and physician-centered decision-making processes [35,36]. Such organizational conditions may influence communication practices, teamwork dynamics, professional engagement, and perceptions of accountability, all of which may affect attitudes toward adverse event reporting.

Correlation analysis demonstrated significant associations between all three EFA-derived factors and perceived benefits of adverse event reporting. Factor 1 (Professional Motivation and Teamwork) and Factor 3 (Staffing Adequacy) were negatively associated with reporting perceptions, whereas Factor 2 (Interprofessional Conflict and Workplace Relationships) demonstrated a positive association. Although some of these relationships may initially appear counterintuitive, previous research has suggested that reporting attitudes are influenced by complex organizational dynamics rather than by isolated organizational characteristics [37,38,39].

One possible explanation is that nurses working in highly cohesive and professionally engaged teams may rely more heavily on informal communication, direct problem-solving, and immediate corrective actions when addressing patient safety concerns. In such environments, formal reporting systems may be perceived as only one component of broader safety management processes rather than the primary mechanism for addressing clinical incidents. Similar observations have been reported in studies emphasizing the role of teamwork, communication openness, and organizational culture in shaping safety-related behaviors [40,41].

The multivariable logistic regression analysis provided additional insight into these relationships. After adjustment for demographic characteristics, Factor 1 (Professional Motivation and Teamwork) remained independently associated with lower odds of favorable perceptions of adverse event reporting, whereas Factor 2 (Interprofessional Conflict and Workplace Relationships) was associated with higher odds of favorable reporting perceptions. Factor 3 (Staffing Adequacy) was not independently associated with the outcome after adjustment.

The persistence of these associations after controlling for age, gender, city, and professional position suggests that organizational characteristics of the work environment may play a more important role in shaping reporting perceptions than individual demographic characteristics. Importantly, none of the demographic variables demonstrated significant associations with the outcome, and adjustment for these variables did not materially alter the observed relationships. This finding addresses the possibility that demographic differences may explain variations in reporting perceptions and supports the relevance of organizational factors as primary explanatory variables [42,43,44].

The positive association observed for the Interprofessional Conflict and Workplace Relationships factor may indicate that environments where communication challenges are more visible may simultaneously increase awareness of patient safety risks and the perceived importance of formal reporting systems. Conversely, nurses working in more harmonious and cohesive environments may be more likely to resolve concerns through direct communication channels before formal reporting becomes necessary. However, because the present study was cross-sectional, these interpretations remain speculative and should be investigated further in future research.

The overall explanatory power of the logistic regression model was moderate, suggesting that additional organizational, leadership, educational, and cultural factors not included in the present study may also influence nurses’ perceptions of adverse event reporting. Previous studies have demonstrated that leadership support, psychological safety, feedback mechanisms, reporting culture, and organizational learning processes may substantially affect reporting behaviors and safety-related attitudes [45,46,47].

The present study contributes to the limited body of evidence examining nursing practice environments and patient safety culture in Central Asian and post-Soviet healthcare systems. While most evidence regarding adverse event reporting originates from Western healthcare settings, considerably less is known about how organizational factors influence reporting attitudes in transitional healthcare systems [48,49]. The findings therefore provide context-specific evidence regarding the organizational dimensions of nursing work environments that may shape reporting-related attitudes among primary healthcare nurses.

From a practical perspective, the findings suggest that strengthening adverse event reporting systems requires more than the implementation of reporting procedures alone. Organizational interventions aimed at improving communication processes, supporting professional engagement, reducing hierarchical barriers, and strengthening teamwork may contribute to more favorable perceptions of reporting systems. Efforts to create psychologically safe environments where nurses can openly discuss patient safety concerns may further enhance engagement in organizational learning and patient safety improvement activities [50,51].

Finally, the findings should be interpreted in light of several methodological considerations. The study utilized a cross-sectional design and self-reported measures, precluding causal inference and creating the possibility of response bias. Furthermore, the identified factor structure reflects the specific organizational context of the study sample and should be interpreted as exploratory until confirmed in independent samples. Consequently, the findings should be viewed as evidence of associations rather than definitive causal relationships.

### Strengths and Limitations

This study has several notable strengths. It included a relatively large sample of nurses from primary healthcare institutions across six major cities in Kazakhstan, which enhanced the diversity of the study population and enabled the analysis of regional variability in nursing practice environments.

The study sample represented a targeted convenience sample recruited through professional, academic, and educational networks rather than as a nationally representative sample of primary healthcare nurses in Kazakhstan. Therefore, the findings should be interpreted primarily in relation to professionally engaged nurses participating in continuing education and professional development activities within urban public primary healthcare settings.

Validated and widely used instruments were applied to assess both the professional practice environment and nurses’ perceptions of adverse event reporting. Both instruments demonstrated acceptable overall internal consistency within the study sample.

Scree plot interpretation and conceptual interpretability were considered during exploratory assessment; however, the final factor retention decision primarily relied on conceptual coherence and interpretability of the extracted factors.

A further strength lies in the use of multiple complementary analytical approaches, including exploratory factor analysis, correlation analysis, and multivariable regression. The combination of these methods allowed for a comprehensive examination of both the structural dimensions of the practice environment and their associations with reporting perceptions.

Several limitations should be acknowledged. The cross-sectional design precludes causal inference. In addition, the use of self-reported data may introduce response bias, including potential social desirability effects.

The sampling strategy included elements of convenience sampling, which may limit representativeness. Participants were recruited through professional and educational networks, potentially resulting in a sample with higher levels of professional engagement. Furthermore, the study focused on nurses working in public primary healthcare settings, predominantly in urban areas, which may limit generalizability to rural regions and private healthcare institutions.

The dichotomization of the outcome variable, applied for logistic regression analysis, may have reduced variability and resulted in some loss of information.

Therefore, caution is required when generalizing the findings to all PHC nurses in Kazakhstan.

Finally, although participation was voluntary and anonymous, and no identifiable data were collected, the mode of survey distribution may have introduced selection bias.

Future research should employ longitudinal designs and include a broader range of healthcare settings to better understand how organizational factors influence adverse event reporting and patient safety culture across diverse contexts.

## 5. Conclusions

This study provides insight into how characteristics of the nursing professional practice environment are associated with nurses’ perceptions of the benefits of adverse event reporting in primary healthcare settings in Kazakhstan. The findings demonstrate that the practice environment functions as a multidimensional organizational context shaping how reporting systems are perceived and utilized in patient safety processes.

Three key dimensions of the practice environment were identified—professional motivation and teamwork, Interprofessional Conflict and Workplace Relationships, and staffing adequacy—reflecting the combined influence of relational and structural factors.

The observed association involving teamwork should be interpreted cautiously and may reflect context-specific communication practices, organizational dynamics, or informal approaches to addressing safety concerns within collaborative clinical environments.

These findings contribute to the limited empirical evidence on nursing practice environments and patient safety culture in Central Asian and post-Soviet healthcare systems. They indicate that strengthening supportive organizational environments, communication processes, and professional engagement may be associated with more favorable perceptions of adverse event reporting.

Given the cross-sectional design, the results should be interpreted as associations rather than causal relationships. Further research using longitudinal designs is needed to better understand how organizational culture, leadership, and system-level factors influence the implementation and effectiveness of reporting systems.

From a practical standpoint, improving patient safety may require an integrated approach that aligns formal reporting mechanisms with informal communication processes. Efforts to strengthen organizational culture, enhance feedback systems, and support nurses’ involvement in clinical decision-making may potentially contribute to the effectiveness and sustainability of reporting practices in primary healthcare.

## Figures and Tables

**Table 1:** Demographic characteristics of nurses across the cities of Kazakhstan.

Characteristics	City						
	Almaty N (%)	Astana N (%)	Shymkent N (%)	Aktobe N (%)	Semey N (%)	Karaganda N (%)	Total N (%)
Age (in years)							
<30	56 (52.8)	5 (4.7)	19 (17.9)	16 (15.1)	5 (4.7)	5 (4.7)	106 (100.0)
30–39	68 (46.3)	4 (2.7)	17 (11.6)	40 (27.2)	7 (4.8)	11 (7.5)	147 (100.0)
40–49	54 (43.9)	7 (5.7)	22 (17.9)	23 (18.7)	8 (6.5)	9 (7.3)	123 (100.0)
≥50	40 (43.5)	4 (4.3)	12 (13.0)	15 (16.3)	15 (16.3)	6 (6.5)	92 (100.0)
Work tenure							
1–5	55 (52.4)	4 (3.8)	14 (13.3)	17 (16.2)	9 (8.6)	6 (5.7)	105 (100.0)
6–15	101 (42.8)	11 (4.7)	35 (14.8)	53 (22.5)	20 (8.5)	16 (6.8)	236 (100.0)
16–25	37 (51.4)	3 (4.2)	12 (16.7)	14 (19.4)	2 (2.8)	4 (5.6)	72 (100.0)
>25	24 (46.2)	2 (3.8)	7 (13.5)	10 (19.2)	4 (7.7)	5 (9.6)	52 (100.0)
Gender							
Female	212 (46.6)	20 (4.4)	67 (14.7)	91 (20.0)	35 (7.7)	30 (6.6)	455 (100.0)
Male	6 (46.2)	-	3 (23.1)	3 (23.1)	-	1 (7.7)	13 (100.0)
Clinical position							
Nurse	182 (45.7)	16 (4.0)	61 (15.3)	83 (20.9)	29 (7.3)	27 (6.8)	398 (100.0)
Senior Nurse	32 (53.3)	4 (6.7)	8 (13.3)	8 (13.3)	5 (8.3)	3 (5.0)	60 (100.0)
Head Nurse	4 (40.0)	-	1 (10.0)	3 (30.0)	1 (10.0)	1 (10.0)	10 (100.0)

**Table 2:** Internal consistency of the study instruments.

Scale	Number of Items	Cronbach’s α
Revised Professional Practice Environment (RPPE)	42	0.87
Reporting of Clinical Adverse Events Scale (RoCAES)	25	0.84

Note: Cronbach’s alpha values ≥ 0.70 indicate acceptable internal consistency.

**Table 3:** Rotated factor loading matrix of the RPPE items.

RPPE Items	Factor 1	Factor 2	Factor 3
I feel a high degree of personal responsibility for the work I do	0.777		
I feel a great sense of personal satisfaction when I do my work well	0.818		
I have challenging work that motivates me to do the best job I can	0.756		
Working in this unit/department gives me the opportunity to gain new knowledge and skills	0.771		
I am motivated to do well because I am empowered by my work environment	0.724		
Working in this environment increases my sense of professional growth	0.748		
There is a lot of teamwork between unit/department staff and doctors	0.733		
Physicians and staff have good working relationships	0.703		
Staff have access to the necessary resources to provide culturally competent care	0.714		
Staff are sensitive to the diverse patient population for whom they care	0.716		
Staff respect the diversity of their health care team	0.701		
Other hospital units/departments seem to have a low opinion of my unit/department		0.756	
Inadequate working relationships with other hospital groups limit the effectiveness of work on this unit		0.709	
When staff disagree, they ignore the issue, pretending it will “go away”		0.761	
Most conflicts occur with members of my own discipline		0.788	
Most conflicts occur with members from other disciplines		0.764	
There are enough staff to provide quality patient care			0.730
We have enough staff to get the work done			0.720

Note: Only factor loadings ≥ 0.40 retained in the final interpretable factor solution are presented. Factor 1—Professional Motivation and Teamwork. Factor 2—Interprofessional Conflict and Workplace Relationships. Factor 3—Staffing Adequacy. Extraction was performed using principal axis factoring, followed by Varimax rotation with Kaiser normalization.

**Table 4:** Spearman correlations between EFA-derived nursing practice environment factors and perceived benefits of adverse event reporting.

Variable	Spearman Correlation Coefficient (r)	*p*
Factor 1—Professional Motivation and Teamwork	−0.562	<0.001
Factor 2—Interprofessional Conflict and Workplace Relationships	0.227	<0.001
Factor 3—Staffing Adequacy	−0.434	<0.001

Note: Spearman correlation coefficients (r) are presented.

**Table 5:** Logistic regression analysis of predictors of perceived benefits of adverse event reporting.

Variable	B	SE	OR (95% CI)	*p*
Factor 1—Professional Motivation and Teamwork	−0.943	0.198	0.389 (0.264–0.575)	<0.001
Factor 2—Interprofessional Conflict and Workplace Relationships	0.567	0.184	1.763 (1.228–2.531)	0.002
Factor 3—Staffing Adequacy	−0.286	0.202	0.751 (0.506–1.116)	0.157
Age	0.018	0.014	1.018 (0.990–1.046)	0.206
Male gender	−0.529	0.771	0.589 (0.130–2.670)	0.493

## Data Availability

The datasets generated and/or analyzed during the current study are available from the corresponding author upon reasonable request.
